# Effectiveness of Genomic Prediction of Maize Hybrid Performance in Different Breeding Populations and Environments

**DOI:** 10.1534/g3.112.003699

**Published:** 2012-11-01

**Authors:** Vanessa S. Windhausen, Gary N. Atlin, John M. Hickey, Jose Crossa, Jean-Luc Jannink, Mark E. Sorrells, Babu Raman, Jill E. Cairns, Amsal Tarekegne, Kassa Semagn, Yoseph Beyene, Pichet Grudloyma, Frank Technow, Christian Riedelsheimer, Albrecht E. Melchinger

**Affiliations:** * Institute of Plant Breeding, Seed Science and Population Genetics, University of Hohenheim, 70599 Stuttgart, Germany; † International Maize and Wheat Improvement Center (CIMMYT), El Batan 56130, Mexico; ‡ United States Department of Agricultural Research Service and Department of Plant Breeding and Genetics, Cornell University, Ithaca, New York 14853; § Nakhon Sawan Field Crops Research Center, Nakhon Sawan 60190, Thailand

## Abstract

Genomic prediction is expected to considerably increase genetic gains by increasing selection intensity and accelerating the breeding cycle. In this study, marker effects estimated in 255 diverse maize (*Zea mays L.*) hybrids were used to predict grain yield, anthesis date, and anthesis-silking interval within the diversity panel and testcross progenies of 30 F_2_-derived lines from each of five populations. Although up to 25% of the genetic variance could be explained by cross validation within the diversity panel, the prediction of testcross performance of F_2_-derived lines using marker effects estimated in the diversity panel was on average zero. Hybrids in the diversity panel could be grouped into eight breeding populations differing in mean performance. When performance was predicted separately for each breeding population on the basis of marker effects estimated in the other populations, predictive ability was low (*i.e.*, 0.12 for grain yield). These results suggest that prediction resulted mostly from differences in mean performance of the breeding populations and less from the relationship between the training and validation sets or linkage disequilibrium with causal variants underlying the predicted traits. Potential uses for genomic prediction in maize hybrid breeding are discussed emphasizing the need of (1) a clear definition of the breeding scenario in which genomic prediction should be applied (*i.e.*, prediction among or within populations), (2) a detailed analysis of the population structure before performing cross validation, and (3) larger training sets with strong genetic relationship to the validation set.

In a hybrid maize breeding program, numerous crosses between inbred lines and testers need to be evaluated in extensive field trials to identify hybrids with greater yield potential in the target environment. Most crosses are discarded after field evaluation due to low general performance. To save resources, it would be advantageous to select inbred lines with high general combining ability by the use of molecular markers, because line performance *per se* is a poor predictor of hybrid performance (Melchinger *et al.* 1998; Hallauer *et al.* 2010). Although a large number of quantitative trait loci (QTL) have been identified, the impact of marker-assisted selection for improving maize hybrid performance in low- and high-yielding environments has been marginal (Tuberosa *et al.* 2007; Araus *et al.* 2008). This is primarily attributed to the small effects of the detected QTL and the fact that many detected QTL are specific to a particular genetic background. Genomic prediction provides an alternative method to use genomic information in breeding decisions. Rather than using only significant marker-trait associations to build up the prediction model, genomic prediction uses all markers simultaneously. The resulting genomic estimated breeding value (GEBV) is the sum of all marker effects (Meuwissen *et al.* 2001). After successful implementation of genomic prediction of breeding values of Holstein and Jersey dairy cattle (Hayes *et al.* 2009a; Goddard and Hayes 2009; Habier *et al.* 2010) and genetic risk of human diseases (Daetwyler *et al.* 2008), it is now beginning to be used in plant breeding programs (Lorenzana and Bernardo 2009).

Using genomic prediction, simulation studies and initial experimental results indicate that grain or biomass yield of maize hybrids can be predicted with high accuracy utilizing one of several different prediction models (de los Campos *et al.* 2009; Crossa *et al.* 2010, 2011; Albrecht *et al.* 2011; González-Camacho *et al.* 2012; Riedelsheimer *et al.* 2012; Zhao *et al.* 2012a). This suggests that rapid increases in rates of genetic gain are possible because prediction accuracy of GEBVs is linearly related to the response to selection. Ideally, a training set composed of genetically diverse individuals, such as different animal breeds (Hayes *et al.* 2009a), would be used for prediction. This would reduce the cost of implementing genomic prediction in breeding programs considerably as the training set would have wide applicability. Nevertheless, more validation experiments are necessary to investigate whether published high prediction accuracies can be applied with as much success in populations different from those in which the marker effects were estimated (Goddard and Hayes 2007). Prediction accuracy of genotypes originating from different populations may be lower than reported in previous studies using genotypes originating from the same population, particularly, if (1) the sample size of the training set is small, (2) broad-sense heritability (*H*) of the trait of interest is low, (3) information from close relatives is not available (Habier *et al.* 2010; Saatchi *et al.* 2011), and/or (4) linkage phases between single-nucleotide polymorphism (SNP) markers and QTL change in sign as suggested for heterotic pools that evolved separately over a long time (Charcosset and Essioux 1994).

The accuracy of genomic prediction is estimated by the correlation between the true breeding value and the GEBV. To date, prediction accuracy has been estimated by evaluating training and validation sets in single and/or the same set of environments. Multienvironment models can benefit from genetic correlations between environments (Burgueño *et al.* 2012). However, it is unknown whether marker effects estimated in a set of environments are predictive of genotype performance in a different set of environments. Furthermore, Riedelsheimer *et al.* (2012) and Saatchi *et al.* (2011) indicated that population structure might affect prediction accuracies. If the genotype set can be subdivided into several clusters or breeding populations that differ in performance level, the correlation between the true breeding value and the GEBV is likely, in part, to be driven by these differences as was also reported for marker assisted selection (Kang *et al.* 2008) and genomic prediction (Albrecht *et al.* 2011; Habier *et al.* 2010; Saatchi *et al.* 2011).

The objectives of this study were to (1) investigate the effects of sample size and number of test environments on prediction accuracy and to evaluate the prediction accuracies in a diversity panel of maize single crosses with the training and validation set drawn from either the same or different environments; (2) examine the prospects for genomic prediction based on testcross data from a diversity panel with a given tester to predict the performance of testcross progeny from segregating biparental populations derived from crosses of lines included or not included in the training set in combination with a different tester in different environments; (3) evaluate prediction accuracy in the presence of population structure; and (4) discuss potential uses for genomic prediction in maize hybrid breeding.

## Materials and Methods

### Genotypes and experimental design

The study used data from two experiments. In Experiment 1, a set of 255 diverse maize inbred lines was used. To summarize in brief, lines were selected to represent the genetic diversity across drought, low-N, soil acidity, and pest and disease resistance breeding programs of the International Maize and Wheat Improvement Center (CIMMYT) and the International Institute of Tropical Agriculture (Wen *et al.* 2011). The lines could be grouped into eight breeding populations based on pedigree information, environmental adaptation, and main breeding target (F. San Vicente, personal communication): lines from the regional CIMMYT breeding program in Zimbabwe (n = 36), from the CIMMYT acid soil tolerance breeding program in Colombia (n = 24), from the CIMMYT insect resistance breeding program (n = 39), from the CIMMYT physiology breeding populations selected for drought tolerance, including the drought tolerant population white (DTPW C9, n= 17) and yellow (DTPY, n = 15) as well as the La Posta Sequía breeding population (n = 39), and from CIMMYT’s subtropical (n =37) and tropical breeding programs (n = 38) in Mexico. For the remaining 10 genotypes, no information on the breeding origin was available. Lines were separated into early- and late-flowering maturity groups and crossed with tester CML312. In total, six trials were conducted in 2010 to 2011 in Mexico and Thailand for both maturity groups.

Experiment 2 comprised five biparental F_2_ populations generated using nine parental lines, four of which were part of Experiment 1. The other five parental lines were distantly related to the lines comprising Experiment 1 (Supporting Information, Figure S1). One hundred fifty test cross progenies were generated by crossing 30 F_2_-derived lines from each cross with tester CML395/CML444 and evaluated in four trials conducted in 2011 in Zimbabwe and Kenya.

All trials were conducted using alpha-lattice designs with two replicates in the dry season under well-watered conditions. Hybrids were evaluated for grain yield, anthesis date, and anthesis-silking interval. Grain yield was recorded in t/ha and adjusted to 12.5% moisture content. Anthesis date was recorded in days after sowing when 50% of plants within a plot shed pollen. Anthesis-silking interval was estimated as the number of days between silking and anthesis date.

### SNP genotyping and marker selection

All 255 inbred lines in Experiment 1 and 30 F_2_-derived lines per population in Experiment 2 (n = 150) were genotyped with the MaizeSNP50 Bead Chip from Illumina, Inc. SNP markers were preprocessed according to the following criteria: (1) less than 5% missing values, and (2) minor allele frequency greater than 5% to exclude SNPs with a high rate of genotyping error and low frequency. A total of 37,403 SNPs met these criteria in Experiment 1 and were subsequently used for validation within Experiment 1. Across Experiment 1 and 2, 18,695 SNP markers were in common after SNP preprocessing. This set of markers was used for validation between Experiments 1 and 2.

### Statistical analysis

#### Variance components and heritability:

Variance components were estimated treating all effects as random effects. Two genotypes were in common across maturity groups.

$$Y_{ijklm}=\mu +g_{i}+e_{j}+ge_{ij}+m{\left(e\right)}_{k(j)}+r{\left(em\right)}_{l(jk)}+b{\left(emr\right)}_{m(jkl)}+\varepsilon_{ijklm}$$, [1]

where *Y* is the mean performance of a certain genotype, *μ* is the overall mean, $g_{i}$ the effect of genotype *i*, $e_{j}$ the effect of the environment *j*, $ge_{ij}$ the interaction between genotype *i* and environment *j*, $m{\left(e\right)}_{k(j)}$ the effect of the maturity group *k* nested in environment *j*, $r{\left(em\right)}_{l(jk)}$ the effect of replicate *l* nested within maturity group *k* and environment *j*, $b{\left(emr\right)}_{m(jkl)}$ the effect of block *m* nested within replicate *l*, maturity group *k* and environment *j*, and $\varepsilon_{ijklm}$ the residual associated with a single plot. The genetic variance among and within breeding populations and clusters (Qst) was estimated by partitioning the genotype effect in model [1] into the effect of the group (breeding population or cluster) and that of the genotype nested within the group. The environment was defined as the year-site combination in which the trials were conducted. It should be noted that individual trials were treated as random samples from the target environment as the purpose of hybrid testing was to predict future performance in farmers’ fields.

Broad-sense heritability (*H*) was estimated across *e* environments and *r* replicates (Hallauer *et al.* 2010):

$$H=\frac{\sigma_{g}^{2}}{\sigma_{g}^{2}+\frac{\sigma_{ge}^{2}}{e}+\frac{\sigma_{\varepsilon }^{2}}{er}}$$, [2]

where $\sigma_{g}^{2}$, $\sigma_{ge}^{2}$, and $\sigma_{\varepsilon }^{2}$ are the genetic, genotype-by-environment, and residual variance components, respectively. *H* was estimated for means over all environments (e = 6) as well as in pairs of e = 4 and e = 2 environments.

On the basis of best linear unbiased estimation, hybrid means were derived in each set of environments (e = 6, 4 or 2) applying model [1] treating the genotype main effects as fixed and all other effects as random.

#### Genetic relationship between lines:

The genetic relationship matrix was estimated by applying method 1 reported by VanRaden (2008). The resulting estimate was divided by two to obtain the kinship among lines. Mean kinship within breeding populations was estimated across all off-diagonal elements. Lines were grouped by specifying the desired number of clusters to n = 5, 10, and 15 using the complete linkage method (Sorensen 1948). Furthermore, the molecular variance among and within breeding populations and clusters (Fst) was assessed applying an analysis of molecular variance.

We investigated the linkage disequilibrium (LD) structure in the largest three breeding populations (*i.e.*, La Posta Sequía, Zimbabwe, and Entomology) by fitting second-order natural smoothing splines onto the scatter plot of LD *vs.* the physical distances between markers on the same chromosome. Only markers with a marker allele frequency >0.05 within the respective breeding population were considered for computing the LD. Furthermore, we investigated the persistence of linkage phases across the three breeding populations following Technow *et al.* (2012). Here, only markers with an allele frequency >0.05 within both breeding populations in the comparison were considered.

#### Genomic prediction:

Hybrid performance was predicted for grain yield, anthesis date, and anthesis-silking interval using ridge regression best linear unbiased prediction (rrBLUP). BLUPs of allelic effects were estimated by assuming that all effects have the same prior distribution and shrinking them toward zero by the same magnitude (Whittaker *et al.* 2000). We define predictive ability [r(ŷ,g)] as the Pearson correlation between the phenotype and the GEBV. The prediction accuracy [r(ĝ,g)] was estimated as the correlation between the true breeding value and the GEBV, obtained by dividing the predictive ability in each run by the square root of *H* of the target trait evaluated in the respective set of environments (e = 6, 4, or 2). Different validation (V) procedures were used to evaluate the effect of different factors on genomic prediction for hybrid performance (Figure S2):

(V1) Effect of sample size and number of test environments: Fivefold cross validation was conducted by subdividing the 255 hybrids of Experiment 1 randomly into five disjoint subsets. One subset was left out for validation whereas the other four subsets were used as training set. This procedure was replicated 20 times, yielding in total 100 runs. Marker effects were estimated in the training set to predict the performance of the validation set evaluated in the same set of environments. The sample size of the training set was varied (n = 204, 156, or 108) as well as the number of environments in which the training and validations set were evaluated (e = 6, 4, or 2).
(V2) Effect of evaluating training and validation sets across different environments: Marker effects were estimated in the training set evaluated in four environments to predict performance of the validation set evaluated in two different environments applying a fivefold cross-validation as described in V1.
(V3) Effect of evaluating training and validation sets with low degree of relationship across different environments, using a different tester: Performance of hybrids generated by crossing 30 F_2_-derived lines with a different tester (Experiment 2) was predicted using marker effects estimated in 255 hybrids (Experiment 1) evaluated in different environments.
(V4) Effect of ‘no’ relationship between training and validation set: Performance of one half of the genotypes in one focal breeding population or cluster was predicted based on marker effects estimated in the remaining breeding populations or clusters. This procedure was replicated 20 times. In each replication a different set of genotypes were placed into the two halves of the focal breeding population or cluster.
(V5) Effect of including relationship between training and validation set: Performance of one half of the genotypes in a focal breeding population or cluster was predicted based on marker effects estimated from a combination of the remaining breeding populations or clusters and the other half of the genotypes in the focal group. This procedure was repeated 20 times as described in V4.
(V6) Prediction based on group means, without the use of markers effects: In each V1 run, the mean of each breeding population or cluster in the training set was used to predict the performance of the genotypes in the validation set. The group mean was estimated across all genotypes of each breeding population and was as such independent of the mean performance of the validation set.

All analyses were performed using the R software version 2.12.2. For estimation of variance components and hybrid means, the *ASREML* package version 3 was used (Butler *et al.* 2009). Breeding values were predicted using the *rrBLUP* package version 2 (Endelman 2011).

## Results

### Variance components and heritability

Mean grain yield of hybrids was 6.88 t/ha in Experiment 1 and 7.02 t/ha in Experiment 2 (Table 1). Mean anthesis date was 71 days after flowering. The early and late maturity group differed in mean anthesis date by 2.6 days (data not shown). The ratio between genotype-by-environment variance and the genetic variance ranged between 0.48 and 1.21, with the greatest values observed for grain yield. *H* across trials was moderate to high for all traits evaluated in Experiments 1 and 2 (0.61-0.85). Within breeding populations, it ranged between 0.34 and 0.84 for grain yield, 0.32 and 0.90 for anthesis date, and 0.31 and 0.71 for anthesis-silking interval (data not shown).

**Table 1:** Mean and standard error of grain yield anthesis date, and anthesis-silking interval, their variance components and broad-sense heritability estimated for 255 hybrids evaluated in six environments (Experiment1) and for 150 testcross progenies of 30 F_2_-derived lines from each population evaluated in 4 environments (Experiment 2)

	Experiment 1			Experiment 2		
Statistic	Grain yield (t/ha)	Anthesis date (days after sowing)	Anthesis-silking interval (days)	Grain yield (t/ha)	Anthesis date (days after sowing)	Anthesis-silking interval (days)
Mean	6.88 ± 0.03	71.35 ± 0.07	2.03 ± 0.03	7.02 ± 0.02	62.28 ± 0.06	0.46 ± 0.11
$\sigma_{g}^{2}$	0.42 ± 0.05	1.66 ± 0.18	0.46 ± 0.06	0.53 ± 0.11	4.87 ± 0.77	0.31 ± 0.06
$\sigma_{ge}^{2}$	0.44 ± 0.03	1.11 ± 0.08	0.22 ± 0.07	0.64 ± 0.10	4.63 ± 0.43	0.22 ± 0.07
$\sigma_{\varepsilon }^{2}$	0.49 ± 0.02	1.39 ± 0.06	2.04 ± 0.08	1.29 ± 0.09	2.47 ± 0.16	1.18 ± 0.08
*H*	0.79	0.85	0.69	0.62	0.77	0.61

$\sigma_{g}^{2}$, genetic variance; $\sigma_{ge}^{2}$, genotype-by-environment variance; $\sigma_{\varepsilon }^{2}$, residual variance; H, broad-sense heritability.

### Genetic relationship and LD

**Figure 1:**
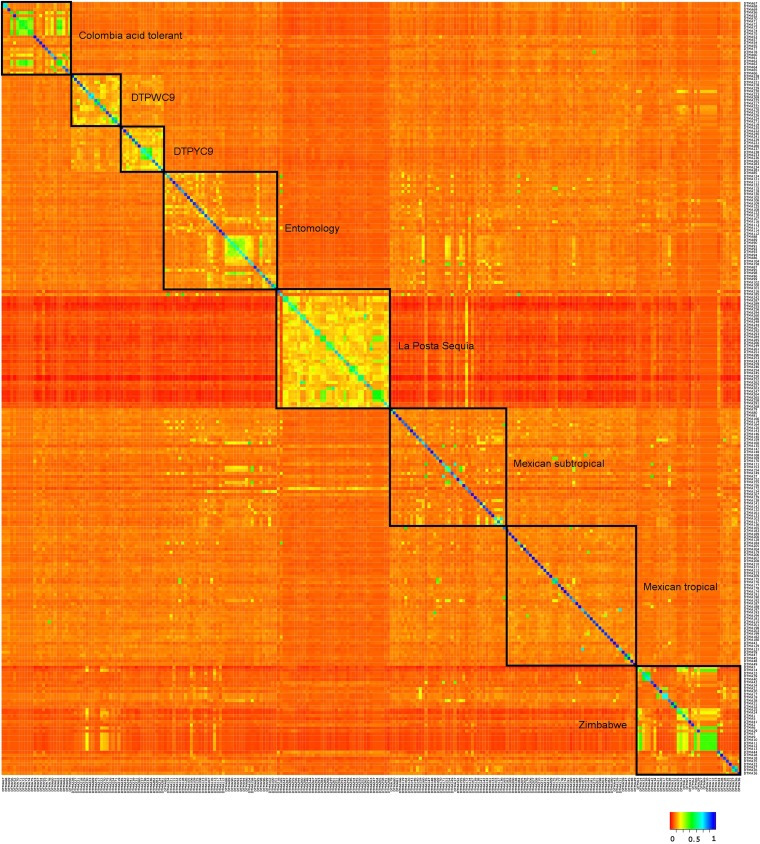
Heat map of the kinship matrix of 255 lines assigned to 8 breeding populations (Experiment 1).

**Figure 2:**
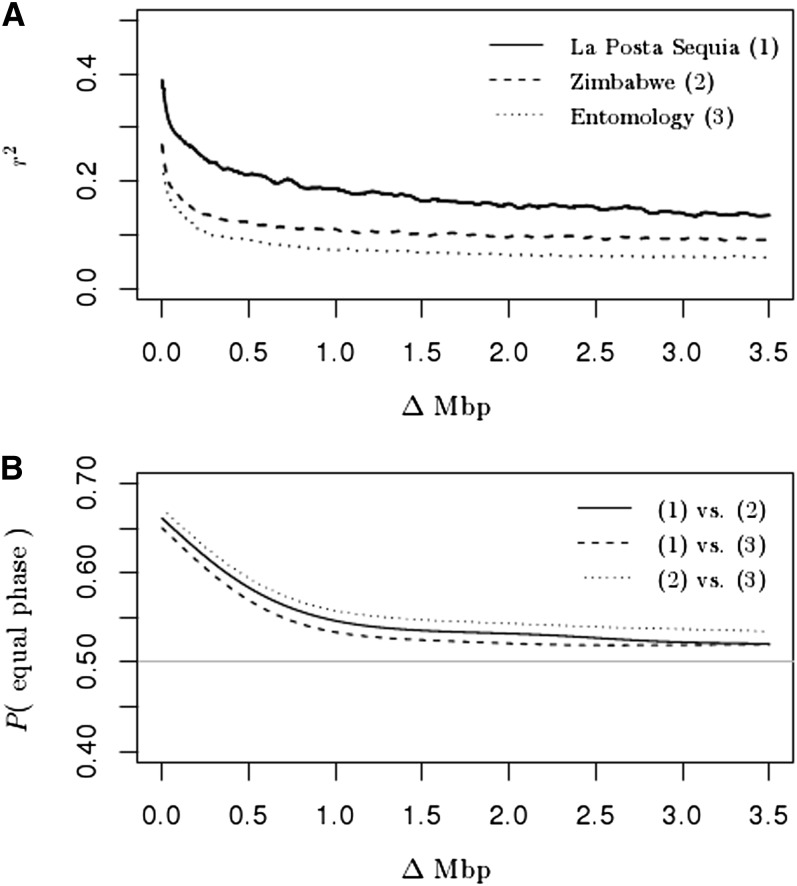
(A) Second-order smoothing spline fits of LD (r^2^) *vs.* the distance in mega base pairs (Mbp) between markers on the same chromosome, within the La Posta Sequía (1), Zimbabwe (2), and Entomology (3) breeding population. (B) Second-order smoothing spline fits of proportion of marker pairs with equal linkage phase *vs.* the distance in marker base pairs between markers on the same chromosome. The horizontal line indicates a linkage phase of 0.5.

Mean kinship within breeding populations of Experiment 1 was between 0.10 and 0.16 for the Colombia acid soil tolerant, La Posta Sequía, DTPW C9, and DTPY C9 breeding populations (Figure 1). For lines derived from the Entomology and Zimbabwe breeding populations, mean kinship was 0.05 and 0.09, respectively. Mean kinship was lowest for the Mexico subtropical and Mexico tropical breeding population. Generally, the relationship within a specific breeding population was greater than among breeding populations. This was particularly true for La Posta Sequía, which had a low kinship to all other breeding populations, as also reported in a previous study using the same genotype set (Wen *et al.* 2011). LD decayed rapidly with physical distance between markers (Figure 2). Furthermore, LD was greater within La Posta Sequía than within the Zimbabwe and Entomology breeding population. The proportion of identical linkage phases across breeding populations was considerably lower than 1 and quickly declined to values close to 0.5 with increasing marker distance.

### Effects of sample size, different environments, and tester on genomic prediction

When genotypes were randomly assigned to the training and validation sets and evaluated in the same environments, predictive ability ranged between 0.30 and 0.45 (Table 2, V1). Predictive ability declined slightly with decreasing number of environments but remained stable when the size of the training set was reduced from 204 to 108 genotypes. Prediction accuracy ranged between 0.43 and 0.50.

**Table 2:** Mean and standard deviation of predictive ability [r(ŷ,g)] and prediction accuracy [r(ĝ,g)] of genomic prediction in Experiment 1 obtained with different number of genotypes (n) and environments (e) in which the training and/or validation set were evaluated

	Training Set		Validation Set		Grain Yield		Anthesis date		Anthesis-silking interval	
	n	e	n	e	r(ŷ,g)	r(ĝ,g)	r(ŷ,g)	r(ĝ,g)	r(ŷ,g)	r(ĝ,g)
Prediction of performance evaluating the training and validation set in the same set of environments										
V1	204	6	51	6	0.44 ± 0.09	0.50 ± 0.10	0.45 ± 0.09	0.49 ± 0.10	0.36 ± 0.13	0.43 ± 0.16
		4		4	0.41 ± 0.11	0.49 ± 0.13	0.42 ± 0.10	0.46 ± 0.11	0.38 ± 0.10	0.50 ± 0.14
		4		2	0.36 ± 0.12	0.49 ± 0.17	0.41 ± 0.12	0.49 ± 0.16	0.31 ± 0.16	0.47 ± 0.30
		2		2	0.39 ± 0.11	0.52 ± 0.16	0.41 ± 0.12	0.49 ± 0.17	0.30 ± 0.15	0.46 ± 0.29
	156	6	100	6	0.44 ± 0.10	0.49 ± 0.11	0.45 ± 0.12	0.49 ± 0.13	0.38 ± 0.12	0.45 ± 0.14
		4		4	0.39 ± 0.13	0.47 ± 0.16	0.43 ± 0.12	0.47 ± 0.13	0.39 ± 0.12	0.51 ± 0.16
		2		2	0.38 ± 0.16	0.50 ± 0.21	0.40 ± 0.14	0.47 ± 0.18	0.31 ± 0.18	0.47 ± 0.32
	108	6	147	6	0.44 ± 0.15	0.50 ± 0.17	0.46 ± 0.11	0.50 ± 0.12	0.37 ± 0.14	0.44 ± 0.17
		4		4	0.39 ± 0.18	0.46 ± 0.21	0.45 ± 0.16	0.49 ± 0.18	0.40 ± 0.17	0.52 ± 0.23
		2		2	0.40 ± 0.14	0.54 ± 0.20	0.45 ± 0.13	0.54 ± 0.17	0.38 ± 0.18	0.57 ± 0.32
Prediction of performance evaluating the training and validation set in the different environments										
V2	204	4	51	2	0.33 ± 0.14	0.39 ± 0.17	0.40 ± 0.16	0.43 ± 0.17	0.32 ± 0.14	0.43 ± 0.19

Genotypes were randomly assigned to the training and validation set under validation schemes V1 and V2

Prediction accuracy of performance in two environments was between 0.47 and 0.49, when based on marker effects estimated in four environments including the two environments of the validation set (Table 2, row 3 in V1). Predictive ability decreased by 0.10 (26%), 0.06 (14%), and 0.04 (9%) for grain yield, anthesis date and anthesis-silking interval, respectively, when the same set of environments were used to predict performance in two different environments (Table 2, V2).

Predictive ability for performance of 30 F_2_-derived lines per population (Experiment 2) was between −0.37 and 0.49 based on marker effects estimated in Experiment 1 (Table 3, V3). Average predictive ability across populations varied around zero.

**Table 3:** Predictive ability for testcross progenies of 30 F_2_-derived lines from each population evaluated in environments (Experiment 2) using marker effects estimated from the 255 inbred lines and phenotypic data of their testcross progenies evaluated in environments (Experiment 1)

Parent 1/2	Breeding Population (Parent 1/2)	GY	AD	ASI
CZL0009*^a^**/*CML539*^a^*	Zimbabwe/Zimbabwe	0.29	−0.03	−0.37
CZL0723/CZL0724	Zimbabwe/Zimbabwe	−0.26	−0.01	−0.10
CZL0723/CZL0719	Zimbabwe/Zimbabwe	−0.20	0.49	0.12
CZL0618/VL0655*^a^*	Zimbabwe/La Posta Sequía	−0.22	0.40	−0.01
CZL074/VL0645*^a^*	Zimbabwe/La Posta Sequía	0.06	0.24	0.08

a These parental lines were included in Experiment 1.

### Genomic prediction among and within breeding populations and clusters

In Experiment 1, predictive ability for performance in a specific group (breeding population or cluster) using marker effects estimated in the other groups, ranged between 0.12 to 0.21 for grain yield −0.01 to 0.23 for anthesis date and −0.03 to 0.02 for anthesis-silking interval with high standard deviations (Table 4, V4). Predictive ability decreased when increasing the number of clusters from 5 to 10 to 15 but was lowest when grouping the genotypes into breeding populations. When 50% of the genotypes in the validation set were included in the training set (Table 4, V5), predictive ability increased for all traits. This increase was greater for anthesis date and anthesis-silking interval than for grain yield.

**Table 4:** Predictive ability for grain yield, anthesis date, and anthesis-silking interval under validation schemes V4-V6 in Experiment 1

	Training Set	Validation Set	Grain yield	Anthesis date	Anthesis-silking interval
V4: Prediction for 50% of the genotypes in one group based on marker effects estimated in all other groups					
5 cluster	177-232	11-39	0.21 ± 0.25	0.23 ± 0.28	0.01 ± 0.20
10 cluster	177-233	11-39	0.23 ± 0.24	0.17 ± 0.36	0.02 ± 0.26
15 cluster	209-230	12-23	0.16 ± 0.23	−0.01 ± 0.23	0.01 ± 0.23
8 populations	216-231	12-19	0.12 ± 0.28	0.02 ± 0.25	−0.03 ± 0.18
V5: Prediction for 50% of the genotypes in one group based on marker effects estimated in all other groups plus the other 50% from the same group					
5 cluster	216-244	11-39	0.31 ± 0.28	0.46 ± 0.21	0.07 ± 0.23
10 cluster	216-244	11-39	0.21 ± 0.24	0.52 ± 0.22	0.16 ± 0.27
15 cluster	232-243	12-23	0.23 ± 0.26	0.39 ± 0.26	0.28 ± 0.28
8 populations	236-243	12-19	0.13 ± 0.25	0.32 ± 0.35	0.03 ± 0.28
V6: Prediction based on group means					
5 cluster	204	51	0.33 ± 0.10	0.21 ± 0.11	0.44 ± 0.09
10 cluster			0.42 ± 0.10	0.31 ± 0.12	0.46 ± 0.10
15 cluster			0.47 ± 0.10	0.37 ± 0.10	0.47 ± 0.11
8 populations			0.50 ± 0.09	0.44 ± 0.09	0.46 ± 0.10

The training and validation sets were evaluated in the same set of environments (e = 6). Genotypes were grouped into 5, 10 or 15 clusters and 8 breeding populations

Breeding populations differed considerably in their mean performance. The difference between the least- and greatest-yielding population was large (1.15 t/ha, Table 5, Table S1) whereas the standard error of means was only between 0.01 and 0.04 (data not shown). Breeding population La Posta Sequía was high yielding, late flowering, and had a shorter anthesis-silking interval (*e.g.*, better flowering synchrony) relative to the other breeding populations. Cross validation methods V1 and V2 (Table 2) partitioned lines from different breeding populations into both the training and validation sets, such that some of the predictive ability was driven by the difference in mean performance (Figure S3). When the mean of each breeding population in the training set was used to predict performance of the genotypes in the validation set (Table 4, V6), predictive abilities were similar to or even greater than in V1, which used markers to predict performance. Even when the genotype set was divided into 15 clusters, genotypes of different breeding populations were placed into the same cluster. This implied that validation in each cluster was conducted across different breeding population means which led to higher predictive ability than when predicting the performance of each breeding population separately.

**Table 5:** Minimum and maximum of grain yield, anthesis date, anthesis-silking interval, and the genetic and molecular variance among ($\sigma_{p}^{2}$) and within ($\sigma_{g(p)}^{2}$) clusters or breeding populations in Experiment 1

	5 Clusters	10 Clusters	15 Clusters	8 Populations
Qst: genetic variance				
Grain yield (t/ha)				
min-max	6.67-7.27	6.67-7.45	6.44-7.45	6.37-7.52
$\sigma_{p}^{2}$	0.05 ± 0.05	0.07 ± 0.05	0.08 ± 0.05	0.11 ± 0.07
$\sigma_{g(p)}^{2}$	0.47 ± 0.04	0.42 ± 0.04	0.41 ± 0.04	0.31 ± 0.04
$\sigma_{p}^{2}/\left(\sigma_{g(p)}^{2}+\sigma_{p}^{2}\right)$	0.096	0.143	0.163	0.262
Anthesis date (days after owing)				
min-max	71.04-72.09	70.39-72.09	70.49-73.09	70.22-72.12
$\sigma_{p}^{2}$	0.13 ± 0.15	0.31 ± 0.24	0.44 ± 0.29	0.28 ± 0.19
$\sigma_{g(p)}^{2}$	3.12 ± 0.28	2.86 ± 0.27	2.81 ± 0.27	1.47 ± 0.16
$\sigma_{p}^{2}/\left(\sigma_{g(p)}^{2}+\sigma_{p}^{2}\right)$	0.040	0.098	0.135	0.160
Anthesis-silking interval (days)				
min-max	1.45-2.17	1.44-2.34	1.41-2.37	1.36-2.31
$\sigma_{p}^{2}$	0.12 ± 0.09	0.12 ± 0.08	0.11 ± 0.07	0.13 ± 0.08
$\sigma_{g(p)}^{2}$	0.57 ± 0.05	0.54 ± 0.05	0.54 ± 0.05	0.35 ± 0.05
$\sigma_{p}^{2}/\left(\sigma_{g(p)}^{2}+\sigma_{p}^{2}\right)$	0.174	0.182	0.169	0.271
Fst: molecular variance				
$\sigma_{p}^{2}$	0.01	0.02	0.02	0.02
$\sigma_{g(p)}^{2}$	0.17	0.16	0.16	0.16
$\sigma_{p}^{2}/\left(\sigma_{g(p)}^{2}+\sigma_{p}^{2}\right)$	0.077	0.099	0.117	0.094

Analysis of genetic variance revealed that dividing the genotype set by breeding populations maximized variance among populations while minimizing variance within populations (Qst; Table 5). For grain yield, the variance among breeding populations explained 26% of the genetic variance while the variance among 15 clusters explained only 16% of the genetic variance. This difference was not observed when estimating the molecular variance (Fst). Here, no matter how many clusters or breeding populations were used to group lines, the variance among groups explained about 10% of the molecular variance.

## Discussion

### Genomic prediction of performance within a diversity panel and testcross progenies of F_2_-derived lines

Within the diversity panel of Experiment 1, the performance of untested genotypes could be predicted, explaining up to 25% of the genetic variance by randomly assigning genotypes to the training and validation set. Much greater prediction accuracies have been reported in previous studies in diversity panels (Crossa *et al.* 2010; Riedelsheimer *et al.* 2012) and segregating populations (Albrecht *et al.* 2011; Zhao *et al.* 2012a,b). Regarding the fact that resources need to be allocated to phenotyping and/or genotyping, we examined the effect of the sample size and the number of test environments on prediction accuracy under validation scheme V1 (Table 2). Contrary to theoretical expectations (Schön *et al.* 2004; Daetwyler *et al.* 2007; Goddard and Hayes 2009), prediction accuracy remained almost constant when reducing the sample size from 204 to 108 and the number of test environments from six to two, which suggests that besides LD and relatedness, other factors, *i.e.*, population structure, contributed to the high prediction accuracy values under validation scheme V1.

By using the diversity panel in Experiment 1 as training set and the F_2_-derived lines of five crosses in Experiment 2 as a validation set, we examined a situation commonly encountered in breeding, where the environments and the tester used in the training set differ from those in the validation set, and where the lines to be predicted have limited relationship with the training set. The predictive abilities observed in validation scheme V3 were disappointing because they varied around zero even for crosses of lines included in the training set (Table 3). According to theoretical results (A. E. Melchinger, unpublished data), the prediction accuracy expected when changing from tester T_1_ in the training set to tester T_2_ in the validation set is obtained as the product of the prediction accuracy with the same tester in the training and validation set and the genetic correlation between the testcross performance of the lines with the two testers. Using the same tester in Experiment 1, predictive ability estimates obtained under validation schemes V1 and V2 were similar using four environments for the training set and two common or different environments for the validation set. Thus, the different environments could not explain the drop in predictive ability observed under V3. Estimates of genetic correlation among two testers were reported to range between 0.6 and 0.9 for grain yield (Bernardo 1991; Melchinger *et al.* 1998). The genetic correlation among the two testers used in the current study is probably of the same order of magnitude but could not be estimated because no testcross data were available with common genotypes. The extent to which line-by-tester interactions contribute to low predictive ability warrants further research.

### Implications of hidden or apparent population structure on genomic prediction

In segregating maize populations (Albrecht *et al.* 2011; Zhao *et al.* 2012b) and different full-sib families in mice (Legarra *et al.* 2008), prediction accuracies were low when the training and validation set comprised genotypes from different crosses or families. Similar to those studies, we investigated whether part of the drop in predictive ability observed under V3 relative to V1 is attributable to population structure. Based on breeders’ information, the 255 lines included in Experiment 1 originated from eight different breeding populations. Mean kinship among breeding populations was low (Figure 1), especially for La Posta Sequía, where LD was higher than within the Zimbabwe and Entomology breeding populations. Differences in LD levels between breeding populations hamper the transferability of marker effects from one breeding population to another, even when the linkage phases are identical. The proportion of identical linkage phases across breeding populations quickly declined with increasing physical distance between markers to values close to 0.5 (Figure 2). Because of differences in LD and linkage phases, marker effects estimated in one breeding population cannot easily be transferred to another, and this at least partly explains the low accuracies observed within breeding populations using marker effects estimated in the other breeding populations (V4 and V5). Interestingly, between two distinct heterotic pools of maize (flint and dent) used for hybrid breeding in Europe, linkage phases decreased less, to a minimum of about 0.6, even though a minimum of 0.5 could have been expected given the long separation of the two pools (Technow *et al.* 2012). The steeper decrease of linkage phases with physical distance between markers in the current study may relate to the smaller sample sizes but also to the fact that the lines in Experiment 1 were developed from rather broad based populations by pedigree breeding accompanied by selection for *per se* and testcross performance with emphasis on different adaptive traits (Wen *et al.* 2011).

Partitioning of the genetic variance across the testcrosses into the variance among and within breeding populations revealed that the former explained 26% of the variance for grain yield (Table 5). This was also reflected by the large difference in the population means of 1.15 t/ha. Reduced genetic distance among lines originating from the same breeding population as compared to those from different breeding populations also was reflected by the heat map of kinship values based on SNP data (Figure 1). Interestingly, in the analysis of molecular variance, the proportion of variance among populations in the total molecular variance was much smaller compared with the subdivision based on the genetic variance of the agronomic traits. Furthermore, the ratio between genetic variance among and within populations was almost three times greater when estimated based on phenotypic data (Qst) than based on marker data (Fst). This finding suggests that SNPs do not fully capture the differences among the lines from different breeding populations. Possibly, selection by breeders results in greater differences at the phenotypic level than reflected by genome-wide markers (Porcher *et al.* 2004; Pujol *et al.* 2008; Whitlock and Guillaume 2009), an observation that warrants further research.

To further investigate the effects of population structure on predictive ability under validation scheme V1, we grouped lines into different numbers of clusters based on the relationship matrix. Including information from relatives into the training set improved within-group prediction substantially for simple traits like anthesis date and anthesis-silking interval, but less so for grain yield. In all instances, predictive ability values including genetic relationship between training and validations sets (V5) were considerably lower compared with V1. Interestingly, when predictions for the lines were solely based on the means of the respective breeding population (V6), we achieved similar or even higher prediction accuracies than with the high-density, SNP-based genomic prediction in V1. Consequently, prediction accuracy across breeding populations resulted mostly from differences in mean performance and less from the relationship between the training and validation set or linkage phases between breeding populations, as also reported in cattle (Habier *et al.* 2010; Saatchi *et al.* 2011). The implications of this result depend on whether previous knowledge of population structure is available and whether one is interested in predicting performance within or among breeding populations. This will be discussed in detail in the next section, *Potential uses for genomic prediction in maize hybrid development*.

### Potential uses for genomic prediction in maize hybrid development

Before incorporating genomic prediction in a plant breeding program, one has to clearly define the breeding scenario in which genomic prediction will be applied. The following scenarios may be differentiated:

#### Training and validation set comprise lines from a diversity panel:

One application of genomic prediction is the performance prediction of new lines in a pedigree breeding program from a large, diverse training set of lines with a low average coparentage with the lines under selection. GEBV accuracy in such populations would result from exploiting LD between high-density markers and QTL controlling the trait. To be effective, this strategy will likely require much larger training sets and denser marker maps than methods depending on close relationships. Simulations for a full sib family indicate that at least 1000 genotypes are required to achieve a prediction accuracy of approximately 0.75 with *H* of the trait of 0.5 (Hayes *et al.* 2009b). Nevertheless, it has to be regarded that greater prediction accuracies are likely to be achieved if the training set is large and includes lines related to the validation set (Habier *et al.* 2010). In six-row barley, Lorenz *et al.* (2012) found little-to-no increase in prediction accuracy when combining distantly related breeding populations to increase the size of the training population. The importance of genetic relationship between training and validation set is discussed in further detail in breeding scenario C.

Prediction accuracy depends on the prediction problem that the breeder is attempting to address. If the goal is to predict within a population that comprises groups of related genotypes with differences in mean performance, results of this study indicate that this can lead to false conclusions regarding the prospects of genomic prediction within groups, which is likely to be the most common application. Prediction accuracy determined with validation scheme V1 in the presence of different groups with different performance levels would only be helpful to breeders if no information on those groups is available, *i.e.*, at the very beginning in breeding for a specific trait like biogas production (Riedelsheimer *et al.* 2012). If no reduction in accuracy is found by reducing the sample size in the training set, this can be taken as an indication for the presence of hidden population structure. In this case, genotyping could be applied to identify groups of related lines. Subsequently, phenotyping a representative sample of lines from each group would be sufficient to determine differences in the performance level of the different groups. If groups are present, it is recommended to take this into account in the validation scheme. Further research is needed on the effect of the number of distinct populations *vs.* the number of lines needed to achieve reliable prediction, as our results show that predictions based on small, highly structured training sets will not achieve useful accuracy. Burgueño *et al.* (2012) showed that for correlated environments, some of the benefits in predictive accuracy come from borrowing information from correlated environments and from using information regarding pedigree and genetic markers. These results indicate that the impact of environmental structure in combination with population structure on prediction accuracy should be considered.

#### Training and validation set are segregating progenies from the same cross:

One application of genomic prediction already used in commercial maize breeding (A. Gordillo, personal communication) is the prediction of performance of double haploid lines which have not been phenotyped, on the basis of a training set derived from the same cross. Similar within bi-parental family predictions were originally envisioned by Bernardo and Yu (2007). This approach would be similar to training and validation within each of the five crosses of Experiment 2, which could not be assessed in the current study due to the low sample size for each population (n = 30). Because multilocation phenotyping is more expensive than one-time genotyping, this approach would allow breeders to generate large full-sib families of doubled haploid lines (*i.e.*, n = 200), phenotype only a small fraction of lines, but large enough to provide reasonably accurate GEBVs (*e.g.*, n = 50), and advance both the best of the phenotyped and unphenotyped full sibs to the next testing stage, based on phenotype and GEBV, respectively. GEBVs are likely to provide moderate accuracy for this application because of the close relationship between the training and validation set and high LD within full-sib families even at low marker density and small population sizes (Wong and Bernardo 2008).

#### Training and validation set include related and unrelated genotypes:

As illustrated by the comparison of predictive ability under validation schemes V4 and V5 including genotypes from the same group in the training set helps to improve predictive ability in the validation set. In maize (Albrecht *et al.* 2011), cattle (de Roos *et al.* 2009) and sheep (Clark *et al.* 2012), it was reported that when the cross-validation scheme allowed for a high degree of relatedness, prediction accuracy increased by 0.26, 0.12, and 0.09, respectively, relative to that achieved across distantly related families. This increase depends on the degree of relatedness between the groups and also whether the LD between markers and QTL is stable across different groups. The latter will depend on the marker density and the breeding history of the groups. If the groups trace back to different races of maize and have been kept separate for a long time and selected with emphasis on different traits, chances are high that LD between adjacent markers is low even with a high marker density. This is similar to the situation in animal breeding, where marker effects estimated in Holstein dairy cattle did not predict accurately GEBVs of Jersey dairy cattle, and *vice versa* (Hayes *et al.* 2009a). An open question in this context is how many groups should be included and how many individuals per group are required to obtain high predictive ability in validation schemes V4 and V5.

#### Recurrent selection with closed synthetic populations of key inbreds:

Another potential application of genomic prediction is rapid-cycle, marker-based recurrent selection in closed populations, like in La Posta Sequía but with a sample size >100, that will serve as sources of inbred lines. The objectives of such a recurrent selection program are to generate an improved population by increasing the frequency of favorable alleles while maintaining sufficient genetic variation for subsequent cycles of selection. One cycle of phenotypic recurrent selection consists of (1) the development of progenies from a population, (2) phenotypic evaluation of the progenies, and (3) selection and recombination of the best selected individuals to form a new population that will form the base material for the next cycle. Genomic prediction would be implemented by genotyping and phenotyping individuals in step (2) and estimating marker effects to predict hybrid performance in the subsequent recurrent cycles and recombine the best lines based on GEBVs alone. Phenotyping would only be used to re-estimate marker effects by evaluating the phenotype of selected parental lines each third recurrent cycle, thus substantially reducing both monetary and time costs associated with phenotyping (Heffner *et al.*, 2009). If these populations were derived from a limited number of parents, high LD between markers and QTL alleles should persist for several cycles of selection, allowing increased genetic gain through acceleration of the breeding cycle with selection based on GEBV alone.

## Supplementary Material

Supporting Information

## References

[bib1] Albrecht T. Wimmer V. Auinger H.-J. Erbe M. Knaak C., 2011 Genome-based prediction of testcross values in maize. TAG 123: 339–350 21505832 10.1007/s00122-011-1587-7

[bib2] Araus J. L. Slafer G. A. Royo C. Serret M. D., 2008 Breeding for yield potential and stress adaptation in cereals. Crit. Rev. Plant Sci. 27: 377–412

[bib3] Bernardo R., 1991 Correlation between testcross performance of lines at early and late selfing generations. Theor. Appl. Genet. 82: 17–21 24212855 10.1007/BF00231272

[bib4] Bernardo R. Yu J., 2007 Prospects for genomewide selection for quantitative traits in maize. Crop Sci. 47: 1082–1090

[bib5] Burgueño J. Campos G. D. L. Weigel K. Crossa J., 2012 Genomic prediction of breeding values when modeling genotype × environment interaction using pedigree and dense molecular markers. Crop Sci. 52: 707–719

[bib6] Butler D. G. Cullis B. R. Gilmour A. R. Gogel B. J., 2009 ASReml-R reference Manual. Department of Primary Industries and Fisheries, Brisbane, Australia

[bib7] Charcosset A. Essioux L., 1994 The effect of population structure on the relationship between heterosis and heterozygosity at marker loci. Theor. Appl. Genet. 89: 336–343 24177851 10.1007/BF00225164

[bib8] Clark S. A. Hickey J. M. Daetwyler H. D. van der Werf J. H., 2012 The importance of information on relatives for the prediction of genomic breeding values and the implications for the makeup of reference data sets in livestock breeding schemes. Genet. Sel. Evol. 44: 1–9 22321529 10.1186/1297-9686-44-4 PMC3299588

[bib9] Crossa J. De Los Campos G. Pérez P. Gianola D. Burgueño J., 2010 Prediction of genetic values of quantitative traits in plant breeding using pedigree and molecular markers. Genetics 186: 713–724 20813882 10.1534/genetics.110.118521 PMC2954475

[bib10] Crossa J. Pérez P. de los Campos G. Mahuku G. Dreisigacker S., 2011 Genomic selection and prediction in plant breeding. J. Crop Improv. 25: 239–261

[bib11] Daetwyler H. D. Villanueva B. Bijma P. Woolliams J. A., 2007 Inbreeding in genome-wide selection. J. Anim. Breed. Genet. 124: 369–376 18076474 10.1111/j.1439-0388.2007.00693.x

[bib12] Daetwyler H. D. Villanueva B. Woolliams J. A., 2008 Accuracy of predicting the genetic risk of disease using a genome-wide approach. PLoS ONE 3: e3395 18852893 10.1371/journal.pone.0003395 PMC2561058

[bib13] de los Campos G. Naya H. Gianola D. Crossa J. Legarra A., 2009 Predicting quantitative traits with regression models for dense molecular markers and pedigree. Genetics 182: 375–385 19293140 10.1534/genetics.109.101501 PMC2674834

[bib14] de Roos A. P. W. Hayes B. J. Goddard M. E., 2009 Reliability of genomic predictions across multiple populations. Genetics 183: 1545–1553 19822733 10.1534/genetics.109.104935 PMC2787438

[bib15] Endelman J. B., 2011 Ridge regression and other kernels for genomic selection with R package rrBLUP. Plant Genome J. 4: 250–255

[bib16] Goddard M. E. Hayes B. J., 2007 Genomic selection. J. Anim. Breed. Genet. 124: 323–330 18076469 10.1111/j.1439-0388.2007.00702.x

[bib17] Goddard M. E. Hayes B. J., 2009 Mapping genes for complex traits in domestic animals and their use in breeding programmes. Nat. Rev. Genet. 10: 381–391 19448663 10.1038/nrg2575

[bib18] González-Camacho J. M. de los Campos G. Pérez P. Gianola D. Cairns J. E., 2012 Genome-enabled prediction of genetic values using radial basis function neural networks. Theoret. Appl. Genet. 125: 759–771 22566067 10.1007/s00122-012-1868-9 PMC3405257

[bib19] Habier D. Tetens J. Seefried F. R. Lichtner P. Thaller G., 2010 The impact of genetic relationship information on genomic breeding values in German Holstein cattle. Genet. Sel. Evol. 42: 1–12 20170500 10.1186/1297-9686-42-5 PMC2838754

[bib20] Hallauer A. R. Carena M. J. Miranda Filho J. B., 2010 Quantitative Genetics in Maize Breeding. Iowa State University Press, Ames, IA

[bib21] Hayes B. J. Bowman P. J. Chamberlain A. C. Verbyla K. Goddard M. E., 2009 a Accuracy of genomic breeding values in multi-breed dairy cattle populations. Genet. Sel. Evol. 41: 1–9 19930712 10.1186/1297-9686-41-51 PMC2791750

[bib22] Hayes B. J. Visscher P. M. Goddard M. E., 2009 b Increased accuracy of artificial selection by using the realized relationship matrix. Genet. Res. 91: 47–60 10.1017/S0016672308009981 19220931

[bib45] Heffner E. L. Sorrells M. E. Jannink J.-L., 2009 Genomic selection for crop improvement. Crop Science 49: 1–12

[bib23] Kang H. M. Zaitlen N. A. Wade C. M. Kirby A. Heckerman D., 2008 Efficient control of population structure in model organism association mapping. Genetics 178: 1709–1723 18385116 10.1534/genetics.107.080101 PMC2278096

[bib24] Legarra A. Robert-Granié C. Manfredi E. Elsen J.-M., 2008 Performance of genomic selection in mice. Genetics 180: 611–618 18757934 10.1534/genetics.108.088575 PMC2535710

[bib25] Lorenz A. J. Smith K. P. Jannink J.-L., 2012 Potential and optimization of genomic selection for fusarium head blight resistance in six-row barley. Crop Sci. 52: 1609

[bib26] Lorenzana R. E. Bernardo R., 2009 Accuracy of genotypic value predictions for marker-based selection in biparental plant populations. Theor. Appl. Genet. 120: 151–161 19841887 10.1007/s00122-009-1166-3

[bib27] Melchinger A. E. Gumber R. K. Leipert R. B. Vuylsteke M. Kuiper M., 1998 Prediction of testcross means and variances among F3 progenies of F1 crosses from testcross means and genetic distances of their parents in maize. Theor. Appl. Genet. 96: 503–512 24710890 10.1007/s001220050767

[bib28] Meuwissen T. H. E. Hayes B. J. Goddard M. E., 2001 Prediction of total genetic value using genome-wide dense marker maps. Genetics 157: 1819–1829 11290733 10.1093/genetics/157.4.1819 PMC1461589

[bib29] Porcher E. Giraud T. Goldringer I. Lavigne C., 2004 Experimental demonstration of a causal relationship between heterogeneity of selection and genetic differentiation in quantitative traits. Evolution 58: 1434–1445 15341147 10.1111/j.0014-3820.2004.tb01725.x

[bib30] Pujol B. Wilson A. J. Ross R. I. C. Pannell J. R., 2008 Are Q(ST)-F(ST) comparisons for natural populations meaningful? Mol. Ecol. 17: 4782–4785 19140971 10.1111/j.1365-294X.2008.03958.x

[bib31] Riedelsheimer C. Czedik-Eysenberg A. Grieder C. Lisec J. Technow F., 2012 Genomic and metabolic prediction of complex heterotic traits in hybrid maize. Nat. Genet. 44: 217–220 22246502 10.1038/ng.1033

[bib32] Saatchi M. McClure M. C. McKay S. D. Rolf M. M. Kim J., 2011 Accuracies of genomic breeding values in American Angus beef cattle using K-means clustering for cross-validation. Genet. Sel. Evol. 43: 1–16 10.1186/1297-9686-43-40 PMC3250932 22122853

[bib33] Schön C. C. Utz H. F. Groh S. Truberg B. Openshaw S., 2004 Quantitative trait locus mapping based on resampling in a vast maize testcross experiment and its relevance to quantitative genetics for complex traits. Genetics 167: 485–498 15166171 10.1534/genetics.167.1.485 PMC1470842

[bib34] Sorensen T., 1948 A method of establishing groups of equal amplitude in plant sociology based on similarity of species content and its application to analyses of the vegetation on Danish commons. Biologiske Skripter 5: 1–34

[bib35] Technow F. Riedelsheimer C. Schrag T. A. Melchinger A. E., 2012 Genomic prediction of hybrid performance in maize with models incorporating dominance and population specific marker effects. Theor. Appl. Genet. 125: 1181–1194 22733443 10.1007/s00122-012-1905-8

[bib36] Tuberosa R. Salvi S. Giuliani S. Sanguineti M. C. Bellotti M., 2007 Genome-wide approaches to investigate and improve maize response to drought. Crop Sci. 47: 120–141

[bib37] VanRaden P. M., 2008 Efficient methods to compute genomic predictions. J. Dairy Sci. 91: 4414–4423 18946147 10.3168/jds.2007-0980

[bib38] Wen W. Araus J. L. Shah T. Cairns J. Mahuku G., 2011 Molecular characterization of a diverse maize inbred line collection and its potential utilization for stress tolerance improvement. Crop Sci. 51: 2569–2581

[bib39] Whitlock M. C. Guillaume F., 2009 Testing for spatially divergent selection: comparing QST to FST. Genetics 183: 1055–1063 19687138 10.1534/genetics.108.099812 PMC2778959

[bib40] Whittaker J. C. Thompson R. Denham M. C., 2000 Marker-assisted selection using ridge regression. Genet. Res. 75: 249–252 10816982 10.1017/s0016672399004462

[bib41] Wong C. K. Bernardo R., 2008 Genomewide selection in oil palm: increasing selection gain per unit time and cost with small populations. Theor. Appl. Genet. 116: 815–824 18219476 10.1007/s00122-008-0715-5

[bib43] Zhao Y. Gowda M. Liu W. Würschum T. Maurer H. P., 2012 a Accuracy of genomic selection in European maize elite breeding populations. Theor. Appl. Genet. 124: 769–776 22075809 10.1007/s00122-011-1745-y

[bib44] Zhao Y. Gowda M. Longin F. H. Würschum T. Ranc N., 2012 b Impact of selective genotyping in the training population on accuracy and bias of genomic selection. Theor. Appl. Genet. 125: 707–713 22481121 10.1007/s00122-012-1862-2

