# Supplementary material for: Effectiveness of Genomic Prediction of Maize Hybrid Performance in Different Breeding Populations and Environments
Source: G3 (Bethesda). 2012 Nov 1;2(11):1427–36. doi: 10.1534/g3.112.003699 (PMC3484673; doi:10.1534/g3.112.003699)
Supplement: Supporting Information [file supp_2_11_1427__index.html]

Supporting Information 

# Effectiveness of Genomic Prediction of Maize Hybrid Performance in Different Breeding Populations and Environments

## Supporting Information for Windhausen *et al.*, 2012

**Files in this Data Supplement:**

- Supporting Information - Figures S1-S3, File S1, and Table S1 (PDF, 729 KB)
- Figure S1 - Cluster of the 255 inbred lines comprising Experiment 1 (black), the 9 parental lines used to establish the five bi-parental F2-populations comprising Experiment 2 (blue) and the tester lines used in Experiments 1 and 2 (red) (PDF, 498 KB)
- Figure S2 - Validation (V) procedures used to evaluate the effect of different factors on genomic prediction for hybrid performance (PDF, 135 KB)
- Figure S3 - Predicted versus actual grain yield using cross validation (V1). (PDF, 125 KB)
- Table S1 - Mean and standard error of grain yield (GY, t/ha), anthesis date (AD, days after sowing) and anthesis-silking interval (ASI, days) in Experiments 1 estimated across and within breeding populations (PDF, 74 KB)
- File S1 - Data sets and R Code (.zip, 4.4 MB)
