# Supplementary material for: Effectiveness of Genomic Prediction of Maize Hybrid Performance in Different Breeding Populations and Environments
Source: G3 (Bethesda). 2012 Nov 1;2(11):1427–36. doi: 10.1534/g3.112.003699 (PMC3484673; doi:10.1534/g3.112.003699)
Supplement: Supporting Information [file supp_2.11.1427_003699SI.pdf]

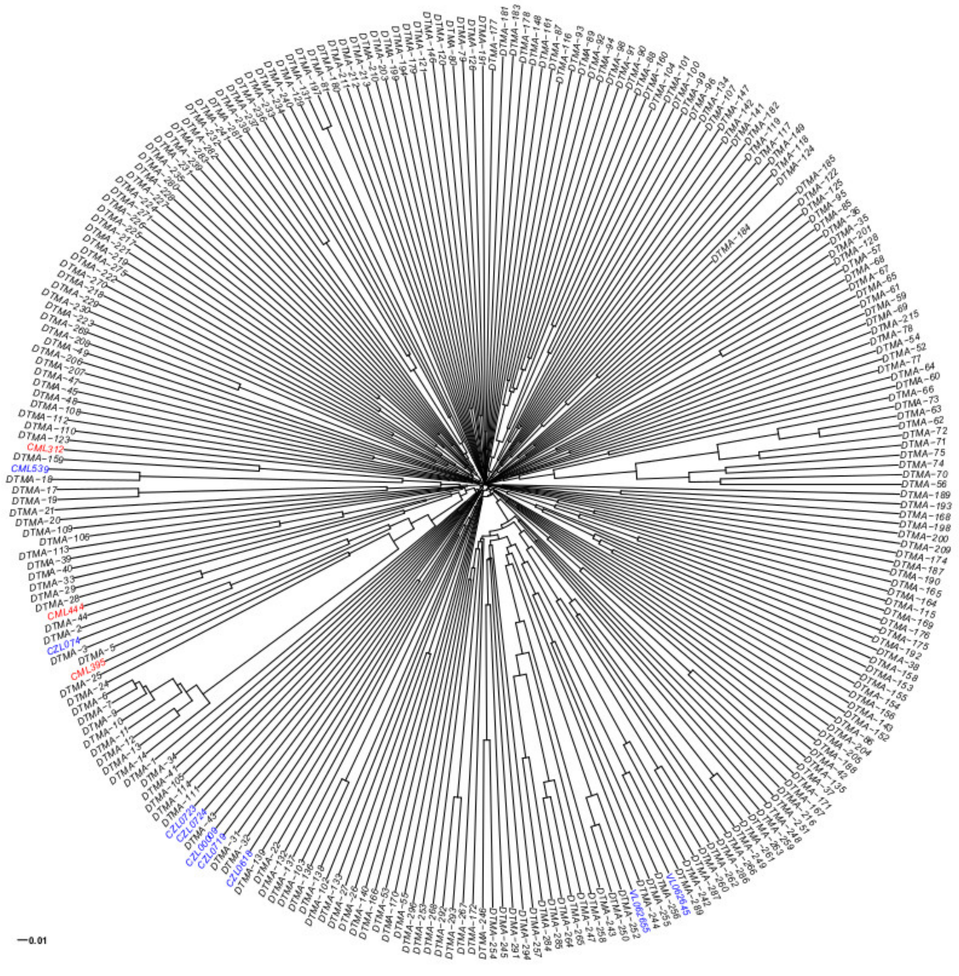

Figure S1 Cluster of the 255 inbred lines comprising Experiment 1(black), the 9 parental lines used to establish the five bi-parental F2-populations comprising Experiment 2 (blue) and the tester lines used in Experiments 1 and 2 (red). The genetic distance between lines was estimated based on modified Rodgers distance (Wright, 1978).

Wright, S., 1978 Evolution and Genetics of Populations. Vol. 6. The University of Chicago Press, Chicago.

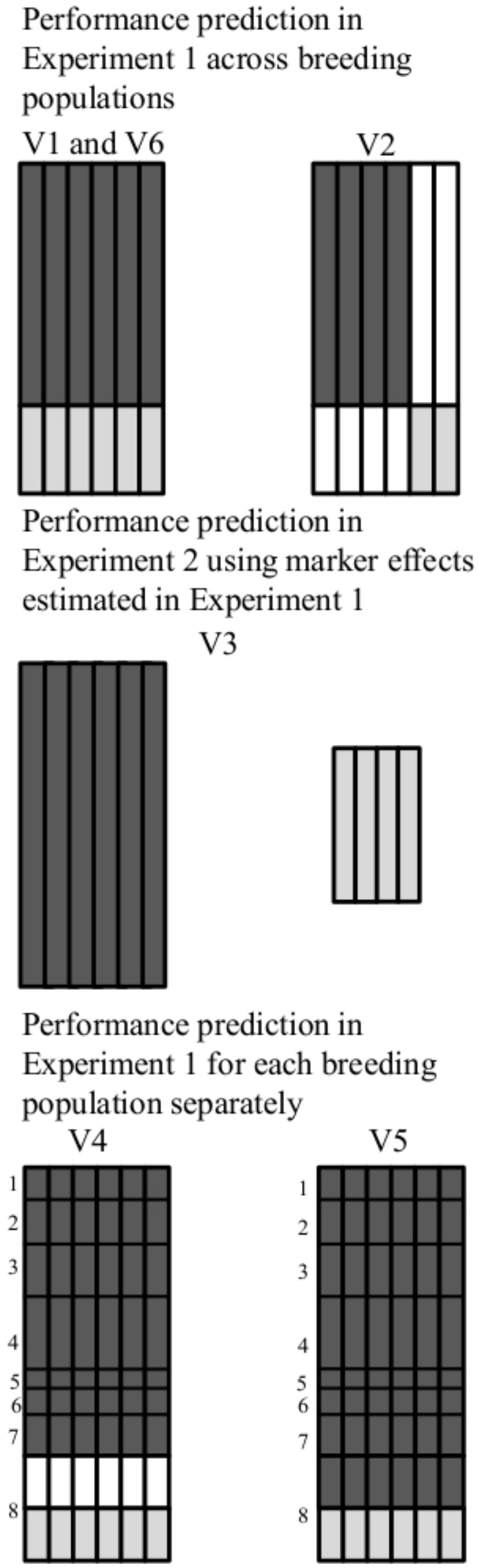

Figure S2 Validation (V) procedures used to evaluate the effect of different factors on genomic prediction for hybrid performance. Marker effects estimated in the training set (dark grey) were used to predict performance in the validation set (grey). Each column represents one environment. Information from white rectangles was not used in cross-validation.

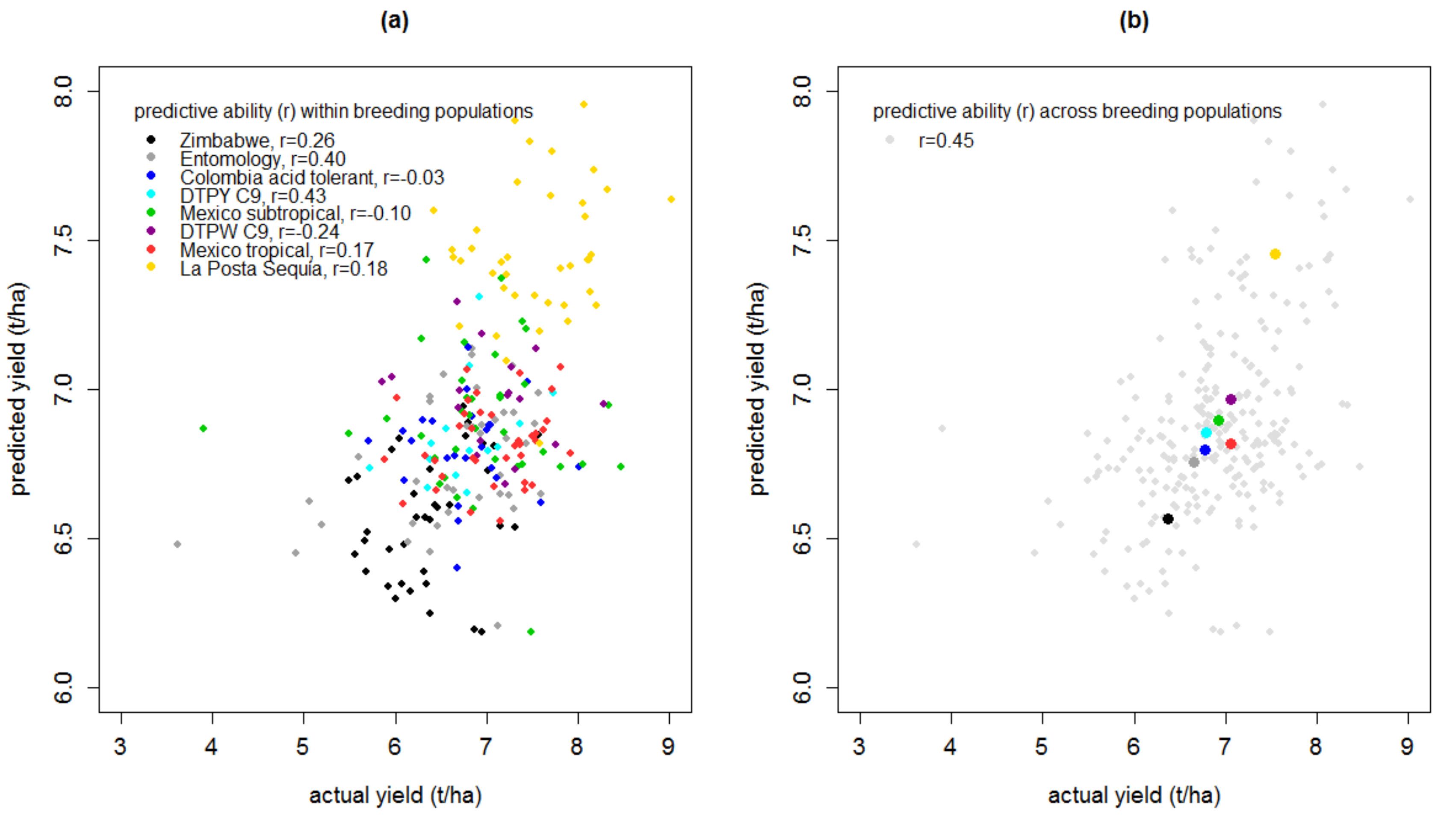

Figure S3 Predicted versus actual grain yield using cross validation (V1). The predictive ability is given within (a) and across (b) breeding populations. The mean performance of each breeding population is highlighted in graphic (b).

File S1

Data sets and R Code

Available for download as a compressed folder at http://www.g3journal.org/lookup/suppl/doi:10.1534/g3.112.003699/-/DC1.

.

Table S1 Mean and standard error of grain yield (GY, t/ha), anthesis date (AD, days after sowing) and anthesis-silking interval (ASI, days) in Experiments 1 estimated across and within breeding populations.

	g[†]	GY	AD	ASI
Across populations	255	6.88±0.03	71.35±0.07	2.03±0.03
Zimbabwe	36	6.37±0.02	72.03±0.09	2.24±0.04
Entomology	39	6.60±0.04	70.59±0.06	2.31±0.03
Colombia acid tolerant	24	6.72±0.02	70.71±0.03	2.52±0.02
DTPY C9	15	6.86±0.01	70.22±0.05	1.48±0.01
Mexico Subtropical	37	6.94±0.04	71.40±0.05	2.14±0.02
DTPW C9	17	7.03±0.02	70.89±0.07	1.65±0.03
Mexico Tropical	38	7.04±0.02	71.32±0.09	2.29±0.03
La Posta Sequía C7	39	7.52±0.03	72.12±0.05	1.36±0.03

[†]Number of genotypes
