# Supplementary material for: Effectiveness of Genomic Prediction of Maize Hybrid Performance in Different Breeding Populations and Environments
Source: G3 (Bethesda). 2012 Nov 1;2(11):1427–36. doi: 10.1534/g3.112.003699 (PMC3484673; doi:10.1534/g3.112.003699)
Supplement: Supporting Information [file supp_2.11.1427_FigureS1.pdf]

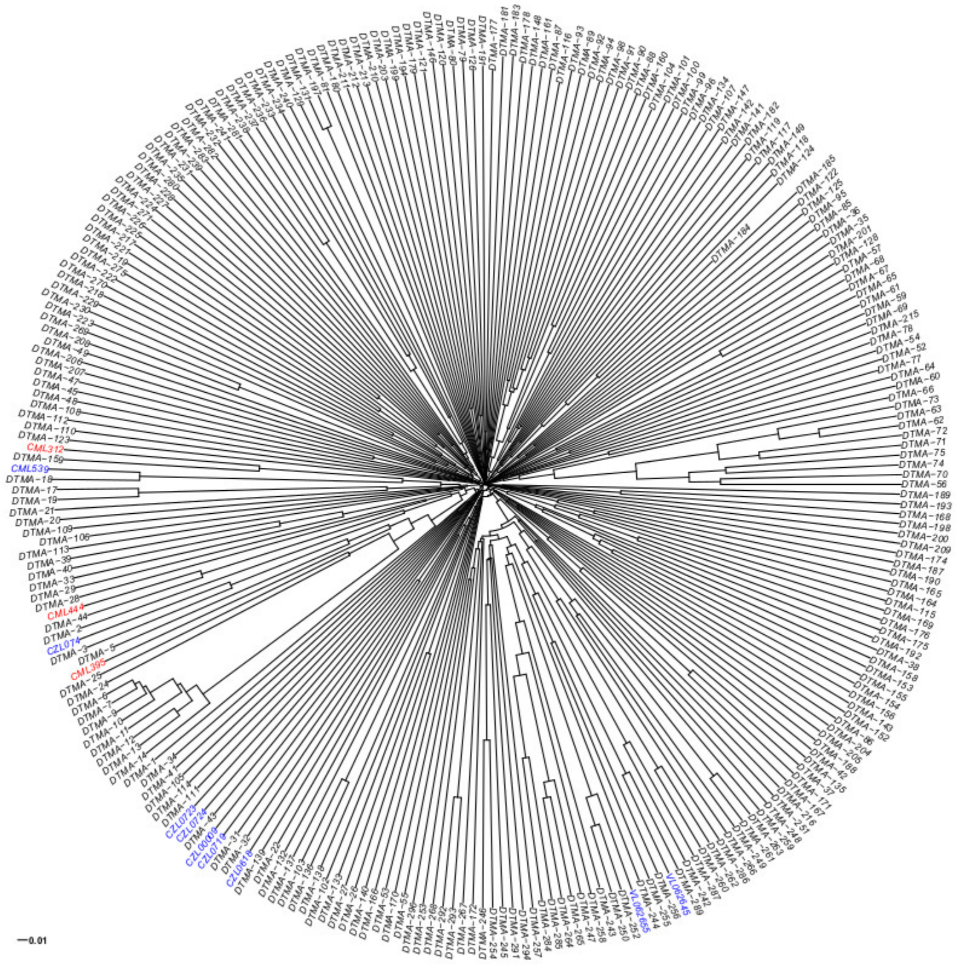

Figure S1 Cluster of the 255 inbred lines comprising Experiment 1(black), the 9 parental lines used to establish the five bi-parental F2-populations comprising Experiment 2 (blue) and the tester lines used in Experiments 1 and 2 (red). The genetic distance between lines was estimated based on modified Rodgers distance (Wright, 1978).

Wright, S., 1978 Evolution and Genetics of Populations. Vol. 6. The University of Chicago Press, Chicago.
