# Supplementary material for: Effectiveness of Genomic Prediction of Maize Hybrid Performance in Different Breeding Populations and Environments
Source: G3 (Bethesda). 2012 Nov 1;2(11):1427–36. doi: 10.1534/g3.112.003699 (PMC3484673; doi:10.1534/g3.112.003699)
Supplement: Supporting Information [file supp_2.11.1427_FigureS2.pdf]

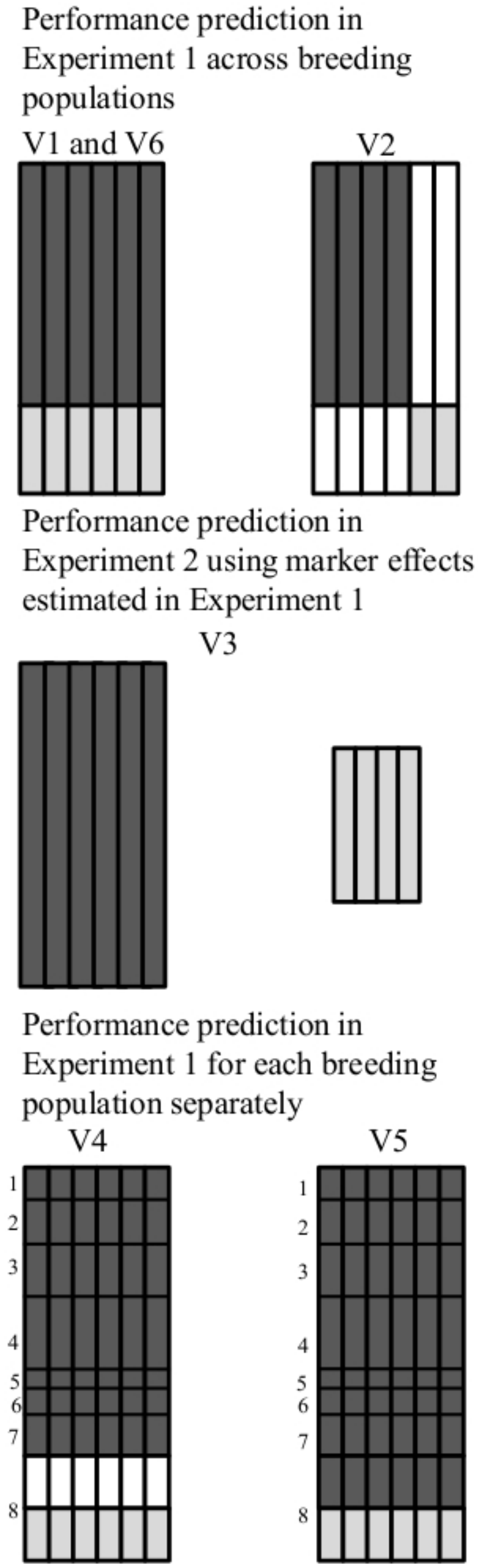


**Figure S2** Validation (V) procedures used to evaluate the effect of different factors on genomic prediction for hybrid performance. Marker effects estimated in the training set (dark grey) were used to predict performance in the validation set (grey). Each column represents one environment. Information from white rectangles was not used in cross-validation.
