# Supplementary material for: Effectiveness of Genomic Prediction of Maize Hybrid Performance in Different Breeding Populations and Environments
Source: G3 (Bethesda). 2012 Nov 1;2(11):1427–36. doi: 10.1534/g3.112.003699 (PMC3484673; doi:10.1534/g3.112.003699)
Supplement: Supporting Information [file supp_2.11.1427_FigureS3.pdf]

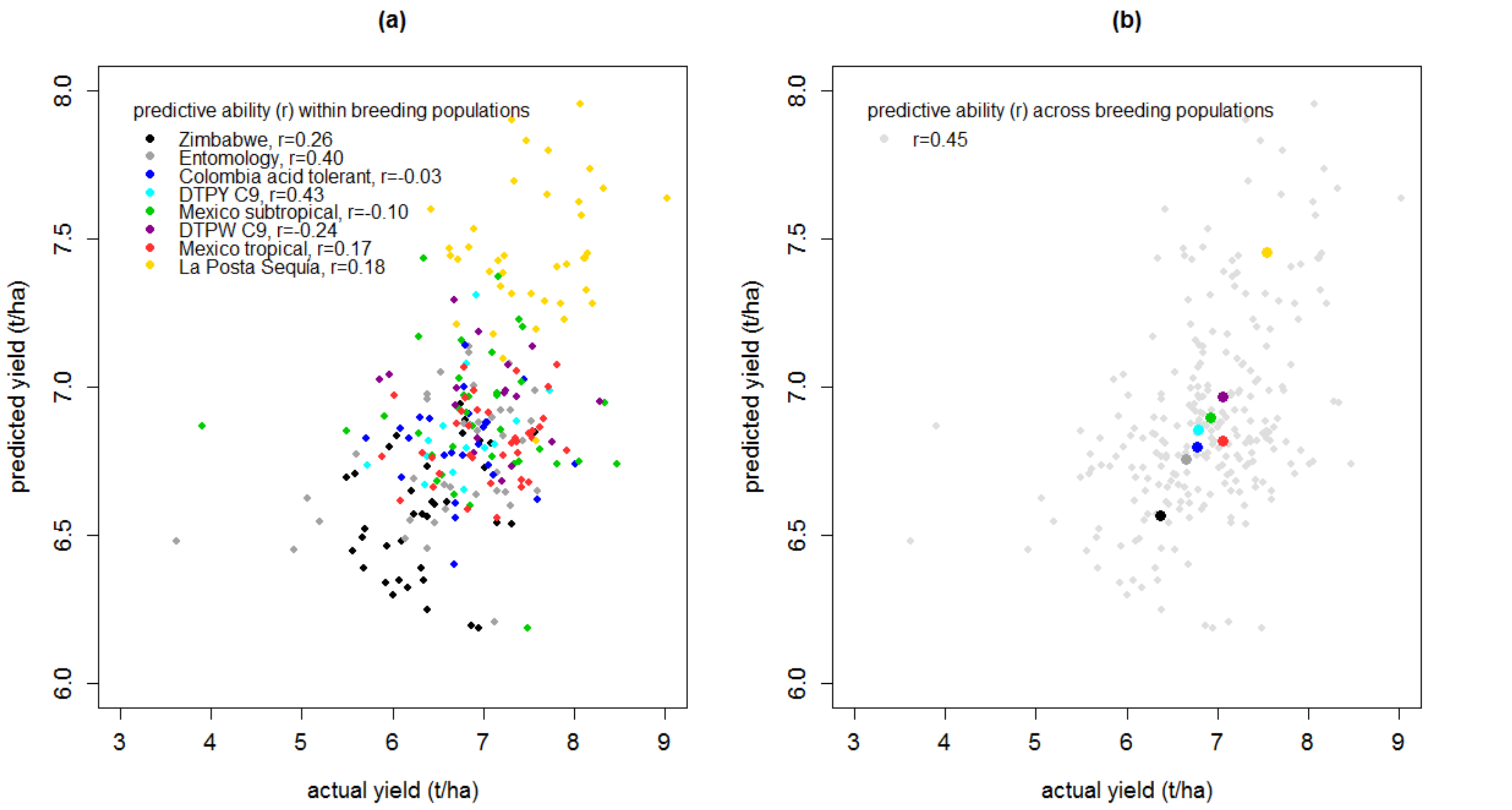

Figure S3 Predicted versus actual grain yield using cross validation (V1). The predictive ability is given within (a) and across (b) breeding populations. The mean performance of each breeding population is highlighted in graphic (b).
