# Supplementary material for: Effectiveness of Genomic Prediction of Maize Hybrid Performance in Different Breeding Populations and Environments
Source: G3 (Bethesda). 2012 Nov 1;2(11):1427–36. doi: 10.1534/g3.112.003699 (PMC3484673; doi:10.1534/g3.112.003699)
Supplement: Supporting Information [file supp_2.11.1427_TableS1.pdf]

Table S1 Mean and standard error of grain yield (GY, t/ha), anthesis date (AD, days after sowing) and anthesis-silking interval (ASI, days) in Experiments 1 estimated across and within breeding populations.

	g†	GY	AD	ASI
Across populations	255	6.88±0.03	71.35±0.07	2.03±0.03
Zimbabwe	36	6.37±0.02	72.03±0.09	2.24±0.04
Entomology	39	6.60±0.04	70.59±0.06	2.31±0.03
Colombia acid tolerant	24	6.72±0.02	70.71±0.03	2.52±0.02
DTPY C9	15	6.86±0.01	70.22±0.05	1.48±0.01
Mexico Subtropical	37	6.94±0.04	71.40±0.05	2.14±0.02
DTPW C9	17	7.03±0.02	70.89±0.07	1.65±0.03
Mexico Tropical	38	7.04±0.02	71.32±0.09	2.29±0.03
La Posta Sequía C7	39	7.52±0.03	72.12±0.05	1.36±0.03

†Number of genotypes
